# Acute aortic dissection with left coronary artery obstruction

**DOI:** 10.1002/ccr3.7719

**Published:** 2023-07-21

**Authors:** Hiroki Uehara, Masaki Okuyama, Yutaro Oe, Takaki Yoshimura, Takahiro Gunji

**Affiliations:** ^1^ Department of Cardiovascular Medicine Kin‐ikyo Chuo Hospital Sapporo Japan

**Keywords:** acute aortic dissection, acute myocardial infarction, coronary angiography, percutaneous coronary intervention

## Abstract

If the electrocardiogram shows ST‐segment elevation in lead _a_V_R_, the complication of aortic dissection must always be assumed.

## Graphical abstract

If the electrocardiogram shows ST‐segment elevation in lead aVR, if coronary angiography shows severe stenosis in the left main trunk, the complication of aortic dissection must always be assumed.

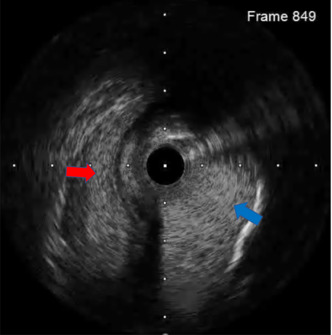

## 1 CASE IMAGES

**FIGURE 1:**
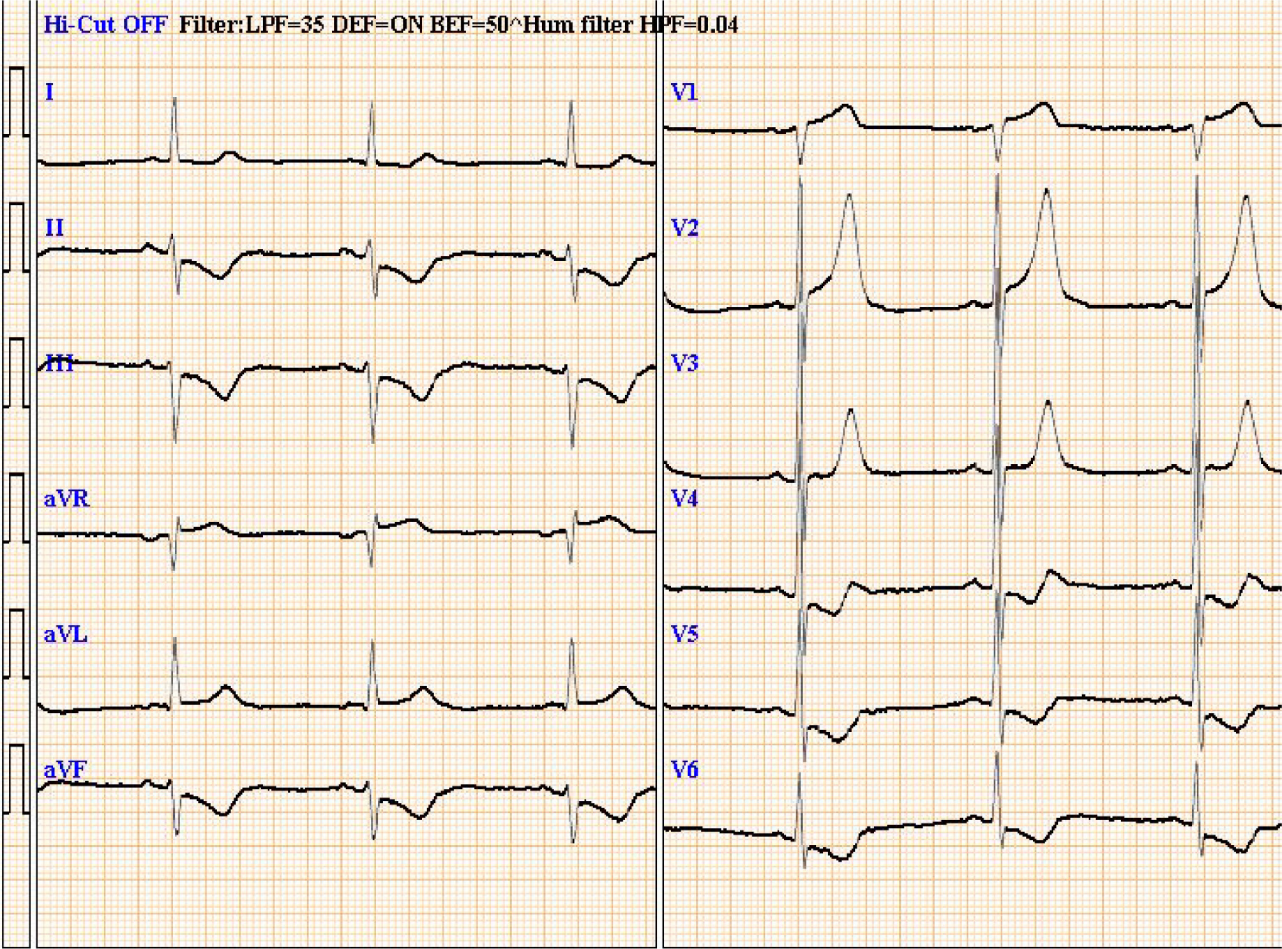
Electrocardiography revealed ST‐segment elevation in lead _a_V_R_ with a mirror image.

**FIGURE 2:**
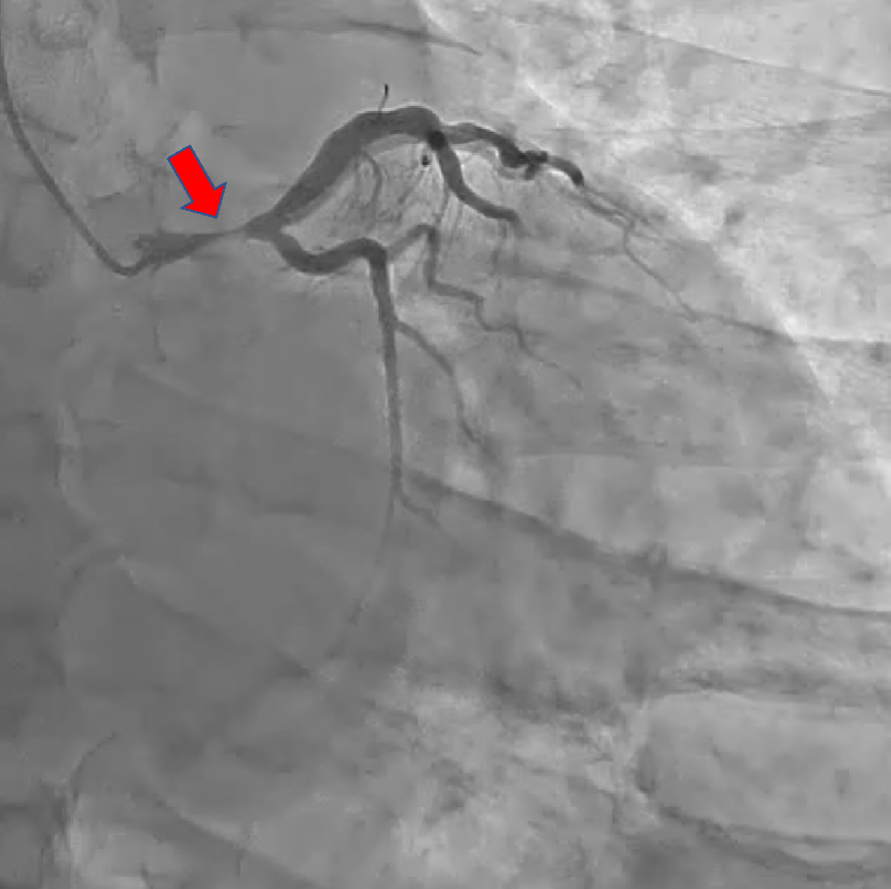
Emergency coronary angiography revealed severe stenosis in the left main trunk (*red arrow*).

**FIGURE 3:**
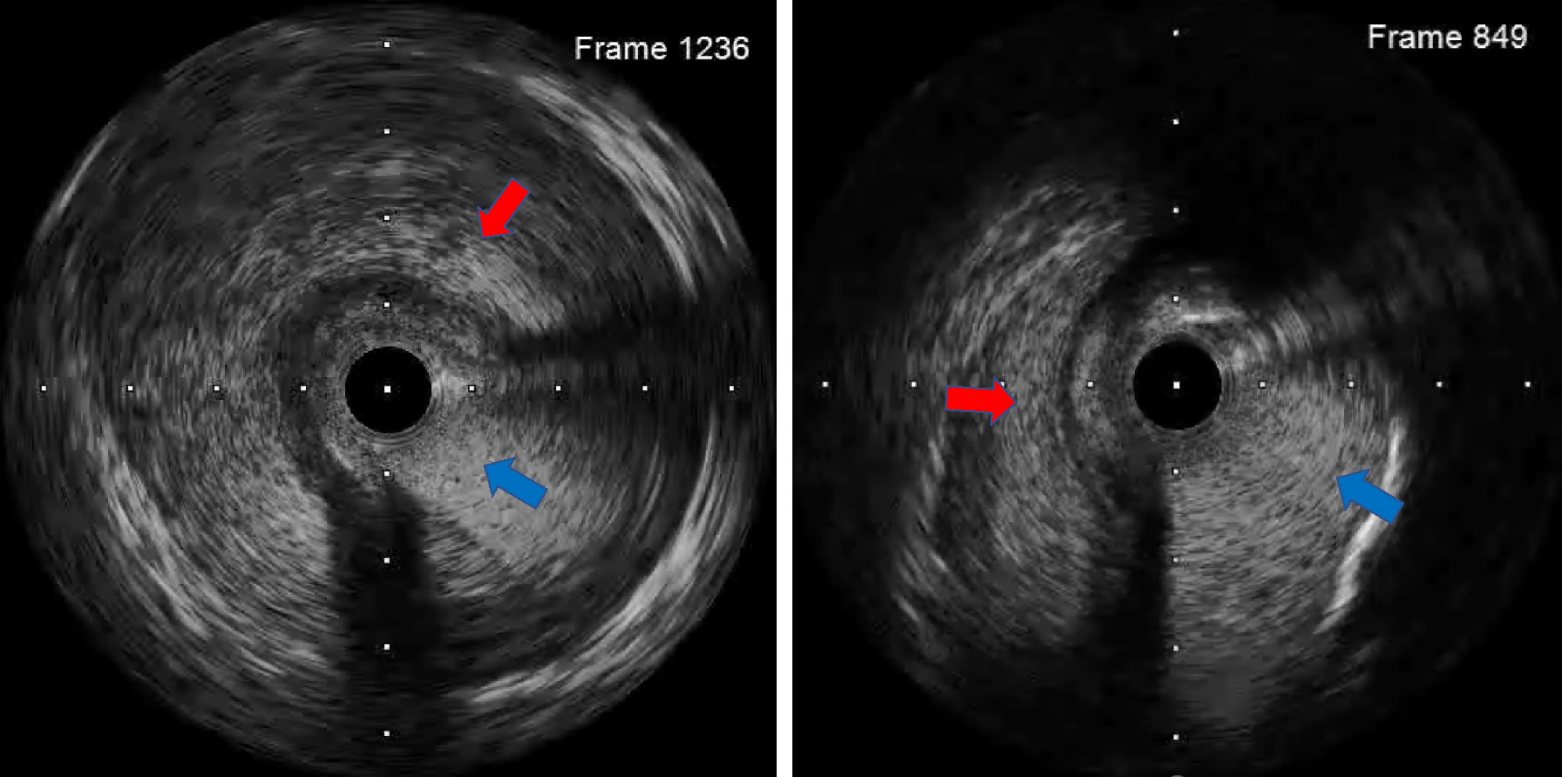
Intravascular ultrasound showed an extensive false lumen (*red arrow*) extending around the true lumen (*blue arrow*).

**FIGURE 4:**
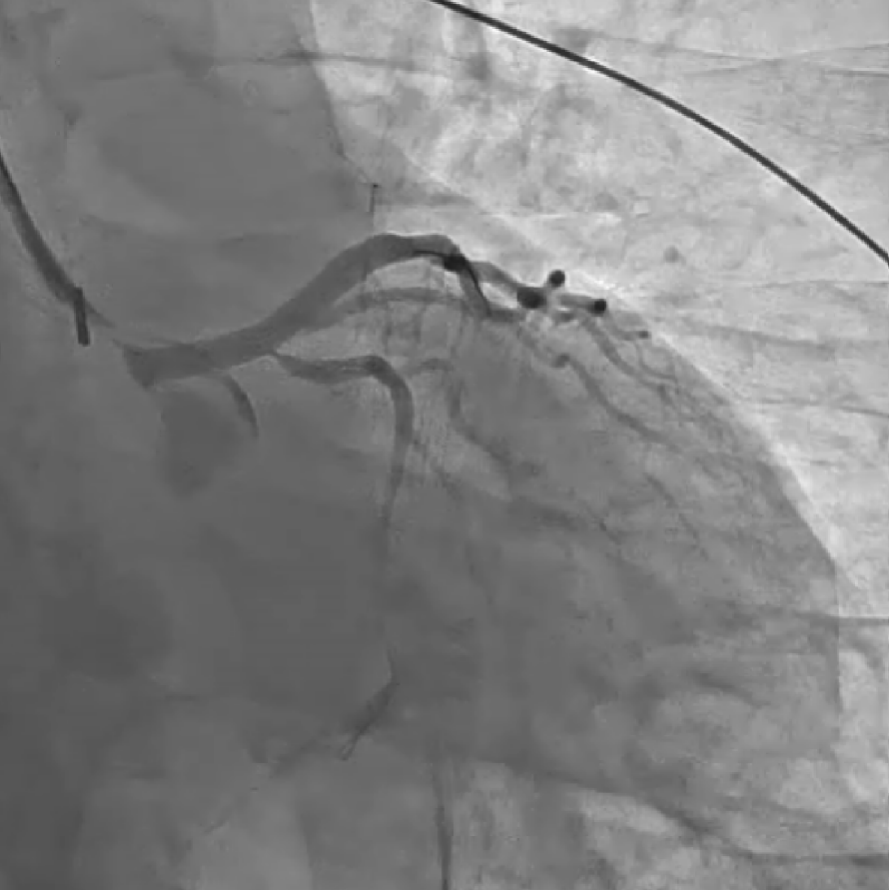
The left main trunk was stented with a 4.00 × 34‐mm drug‐eluting stent.

**FIGURE 5:**
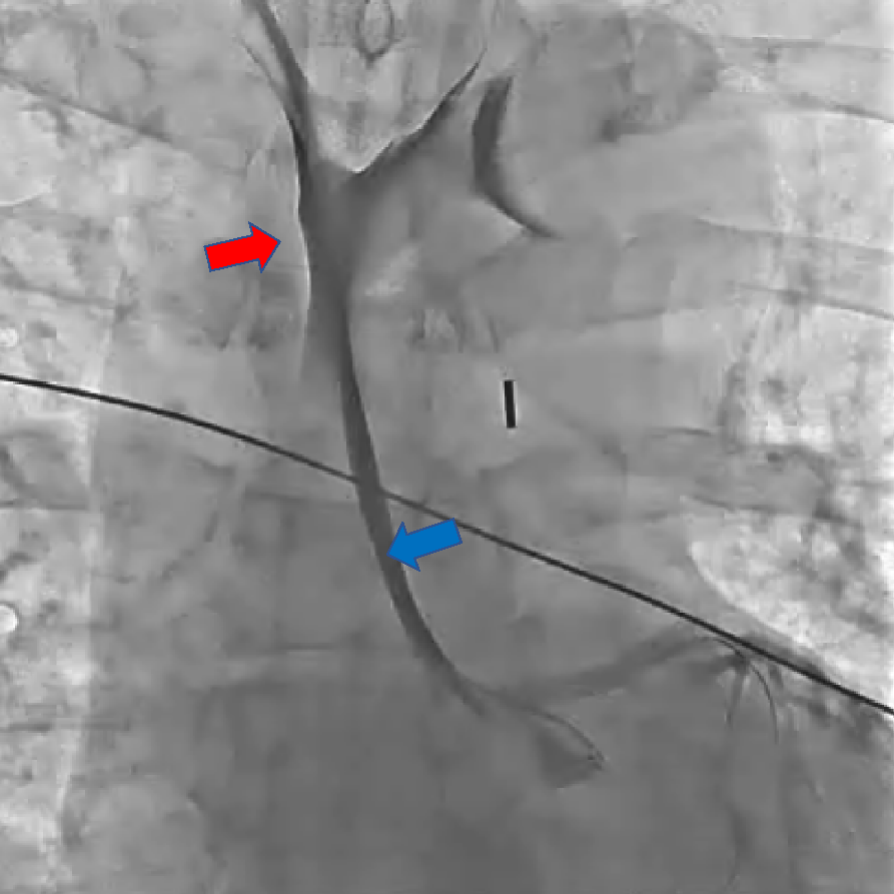
Angiography of the coronary arteries showed antegrade contrast of the ascending aorta (*blue arrow*) compressed by the false lumen (*red arrow*).

A‐54‐year‐old Japanese man with sudden chest discomfort visited our hospital. Electrocardiography revealed ST‐segment elevation in lead _a_V_R_ with a mirror image (Figure 1). Echocardiography showed no evidence of pericardial effusion, ascending aortic flap, or aortic regurgitation, but wall‐motion impairment of the anterior and lateral walls of the left ventricle was observed. Emergency coronary angiography revealed severe stenosis in the left main trunk (Figure 2). The patient suddenly went into cardiopulmonary arrest, and percutaneous cardiopulmonary support was initiated. We started a 7‐French sheath via the right radial artery and engaged a 6‐French guiding catheter. Intravascular ultrasound showed an extensive false lumen extending around the true lumen (Figure 3). The left main trunk was stented with a 4.00 × 34 mm drug‐eluting stent. (Figure 4). Angiography of the coronary arteries showed antegrade contrast of the ascending aorta compressed by the false lumen (Figure 5). Repeated echocardiography showed no evidence of ascending aortic flap. However, the diagnosis of acute aortic dissection with left coronary artery obstruction was made from angiography and IVUS. His heartbeat never resumed, and surgery treatment could not be performed, and his life could not be saved. It was not possible to record CT images or echocardiographic videos.

In Type A acute aortic dissection, ST‐segment elevation in lead _a_V_R_ is a myocardial infarction complication, and it is the strongest predictor of in‐hospital death.^1^ If electrocardiography shows this change, the complication of aortic dissection must always be assumed.

## AUTHOR CONTRIBUTIONS

**Hiroki Uehara:** Conceptualization; data curation; formal analysis; funding acquisition; investigation; methodology; project administration; resources; software; supervision; validation; visualization; writing – original draft; writing – review and editing. **Masaki Okuyama:** Supervision. **Yutaro Oe:** Supervision. **Takaki Yoshimura:** Supervision. **Takahiro Gunji:** Supervision.

## CONFLICT OF INTEREST STATEMENT

The authors report no potential conflicts of interest associated with this research.

## CONSENT

The patient's written informed consent for the publication was obtained by his family, and his identity has been protected.

## Data Availability

Data sharing is not applicable to this article as no new data were created or analyzed in this study.
